# Development of a checklist to assess potentially effective components in combined lifestyle interventions for children with overweight or obesity

**DOI:** 10.1371/journal.pone.0289481

**Published:** 2023-09-28

**Authors:** Jenneke J. E. H. Saat, Gerdine A. J. Fransen, Elke Naumann, Koos van der Velden, Willem J. J. Assendelft

**Affiliations:** 1 Department of Primary and Community Care, Academic Collaborative Centre AMPHI, ELG 117, Radboud University Medical Centre, Nijmegen, the Netherlands; 2 Research Group Nutrition, Dietetics and Lifestyle, HAN University of Applied Sciences, Nijmegen, The Netherlands; 3 Department of Primary and Community Care, ELG 117, Radboud University Medical Centre, Nijmegen, the Netherlands; St John’s University, UNITED STATES

## Abstract

**Background:**

In the Netherlands, Combined Lifestyle Interventions (CLIs), offered in primary care, aim to reduce the number of children with overweight or obesity. CLIs are carried out by a multidisciplinary team and focus on dietary advice and guidance, exercise and behaviour change. These CLIs are not uniformly designed and vary in protocols to suit the local circumstances. Due to the variation in content of CLIs it is difficult to investigate their effectiveness. To enable a proper evaluation of CLIs, we first need to unravel the ‘black boxes’ of CLIs by identifying the various potentially effective components.

**Methods:**

First of all we identified potentially effective components in literature. Subsequently we organized an online consultation with experts with diverse backgrounds and asked if they could add potentially effective components. These components were then assembled into a checklist meant to determine the presence or absence of potentially effective components in CLIs for children.

**Results:**

42 experts participated. We identified 65 potentially effective components for CLIs for children with overweight or obesity that we categorized into three themes: content, organisation and implementation.

**Conclusions:**

Based on literature and expert opinions we developed a practical 65-item checklist to determine the presence of potentially effective components in a CLI. This checklist can be used in the development of CLIs as well as evaluation of CLIs.

## Introduction

The alarmingly high prevalence of children with overweight or obesity [1]) and its perceived lower health-related quality of life (HRQOL) [2] indicate the urgency to reduce this problem. Combined lifestyle interventions (CLIs) are used to prevent and treat childhood overweight and obesity and focus on dietary advice and guidance, exercise and behaviour change [3–6]. Because CLIs need to suit local circumstances of the community [7] CLIs differ in, for example, their method of delivery (group versus individual meetings, behaviour change techniques), indication (overweight or obese), start of CLI (immediately after diagnosis or only after period of self-management), duration, intensity (frequency of meetings) and the professional background of involved implementors (professionals responsible for implementing the CLI). These features can be seen as components of a CLI. The framework of the Dutch National Institute for Public Health and the Environment [8] defines ‘effective components’ as: those parts of an intervention that ensure that a specific intervention has the desired effect. Goals are achieved and contribute to an improvement or solution of the problem. Additionally, an effective component that likely contributes to the achievement of the objective, but on which the evidence is still limited is defined as: ‘potentially effective component’ [9].

Although CLIs are frequently implemented, CLIs are ‘black boxes’ of which we do not know what components determine the effectiveness. There is an increasing focus on studying potentially effective components of CLIs for the general adult population [10,11], adults with a low social economic status [12] and adults with extremely increased weight-related health risk [13].

However, to the best of our knowledge, the potentially effective components of CLIs for children have not yet been studied. Understanding the effective components of CLIs for children contributes to the improvement of existing and the development of new effective CLIs that promote long-term healthy behaviour in children with overweight or obesity.

The aim of this study was to explore potentially effective components and develop a practice-oriented checklist, based on the literature and with input of a broad group of experts. These experts are involved as implementors, researchers or policy makers of CLIs for children with overweight or obesity. We asked them to name additional potentially effective components than described in literature, based on their working experience. In practice or for research purposes, this checklist can subsequently be used to determine the presence of potentially effective components in existing CLIs for children with overweight or obesity or in new CLIs that may be developed in future.

## Materials and methods

We first conducted a literature search and synthesis to extract potentially effective components. We aimed at children from 4–18 years with overweight or obesity. Next, selected experts were consulted to add potentially effective components that were not found in our literature search. Thereafter, we constructed a concept checklist to determine the presence or absence of potentially effective components in the protocol of a CLI and then checked the list on useability. Finally, we adapted the checklist into a final version.

### Ethics statement

This study was reviewed by the Radboudumc Human Ethics Committee Nijmegen (Reference 2017–3175). The committee decided that the Medical Research Involving Human Subjects Act (WMO) did not apply for this study. Before entering the study, written informed consent of all participants, who were professionals aged 18 years and older, was obtained, because a written process suits (the context, design, and participants) the study.

### Literature search and synthesis

A literature search was conducted in October 2016 in the database PubMed, restricted from publication year 2000 to more recent and available in English. Search terms used were related to overweight, obesity, children, combined lifestyle intervention, effective components, and their synonyms. For the ‘effective component’, we used the following search terms: core components, essential elements, and success factors. Titles and summaries were screened on information regarding effective treatment or effective components of CLIs targeting children with overweight or obesity. If a study seemed useful, the full text article was read to make a final decision. In addition, references of relevant publications were used to find additional relevant articles. We repeated these steps until saturation was reached regarding described effective components.

Potentially effective components were extracted from the articles by marking the components by three researchers independently (J.S., G.F. & E.N.). Components were, together with their description and reason for effectiveness, given a code and added to a code table, using Atlasti (version 9.1.6). Identified codes were used for subsequent articles. Saturation resulted in a final list of potentially effective components from the literature.

### Expert panel

To identify additional effective components not retrieved from our literature search, experts in the following three sectors were recruited: the field of research or evaluation of CLIs, development or coordination of CLIs, or implementation of a CLI. We pursued purposive sampling, based on professional background, work setting and work region. Between September and December 2018, 103 experts were invited by email to participate in an online consultation. Invited experts were asked to forward the invitation to people who, according to their opinion, could also contribute.

### Expert consultation

An online questionnaire was sent to the experts using the programme Lime Survey (version 3.0). The experts were asked for personal characteristics, such as professional background, work setting and work region. Then, the randomly ordered concept list of potentially effective components arisen from the literature search was presented with an explanation of the components and reference(s). The experts were asked: ‘Do you think the list contains all the potentially components that you think are effective in CLIs for children with overweight or obesity?’. If the answer was ’No’, they were asked to add new components, preferably with an explanation, and (if available) references to literature. They could also provide literature suggestions or comments to the components already listed. At the end of the questionnaire, experts could give any further suggestions and comments to the study in open text. The first author (J.S.) compared components that were added by the experts with the components already available from the literature. If there were ambiguities regarding an added component (for example when a proposed component had similarities with a component already in the concept list) J.S. contacted the expert who had suggested to add this component for clarification. Recommended references were explored, following the same procedure for extracting components as described in Literature Search and synthesis. J.S. made suggestions for adding new components which were discussed in the core research team (J.S., G.F. & E.N.) that constructed the final list.

### Instrument construction

After the expert consultation, we translated all components of the final list into a pre-final checklist, with options (depending on the type of component) to state the absence or presence or to state characteristics (e.g., number of meetings, duration of the programme).

We tested the pre-final checklist on its usability and reliability to optimize the descriptions and the order of components, and to determine whether all possible answer options were present. Since components that define a CLI are described in a protocol or manual of the CLI, we collected protocols of different existing CLIs with sufficient variety, to assess the pre-final checklist. Of six CLIs implemented in the region of the research team’s network, the protocol was available via the coordinator of the relevant CLIs. Each CLI protocol was assessed by two researchers (G.F., E.N., J.S.) independently. Two researchers each assessed two or three CLIs in a varying composition. After each assessment, the two researchers compared their scoring on each component, noted the percentage of agreement, and (regardless of whether the score was different) discussed difficulties in scoring the absence or presence of the different components. In case they experienced difficulties in scoring the component into ‘absent’ or ‘present’, the descriptions were discussed and, when necessary, modified. Unclear wording was optimized, and, in some cases, components were split or combined. The improved version of the checklist was then used in the assessment of a subsequent CLI protocol.

### Repeated literature search

Finally, in 2023 we repeated the literature search and synthesis to explore if updated scientific literature and guidelines could provide additional potentially effective components to the final checklist’. For this, we followed the same procedure as described in the subparagraph ‘Literature search and synthesis’. In addition, for all references included in our first search, citation searching was performed using the citation search function on Google Scholar.

Additional potentially effective components were, if applicable and agreed by three researchers (J.S., G.F. & E.N.), added to the results of our first literature search and to our checklist’.

### Results

#### Literature search

The literature search and synthesis resulted in a list with 23 potentially effective components, see ‘Table 1 Potentially effective components described in literature’.

**Table 1:** Potentially effective components described in literature.

1	Healthcare professionals realise awareness in children and their parents	[14–17]
2	Healthcare professionals enhance motivation	[14,15,17–**19]**
3	Discuss the exemplary role of the parent	[15,**16,20,21**]
4	Support parents in raising children	[15**,^16^**]
5	Adapt to the culture of the child	[**16**,^22^]
6	Adapt to the income of the parents for feasibility and affordability	[15]
7	Create a tailored treatment plan (that fits into daily life of the whole family)	[3,15,**16**,^18^**,^19^,^21^**,**23]**
8	Encourage to spend less time on watching television	[6]
9	Discuss the influence of the physical environment of the child	[24]
10	Discuss the influence of the social environment of the child	[18,24]
11	Discuss and optimize sleep patterns and duration of the child	[24]
12	Ensure enough dosage in length of sessions	[3,**23**,^25^,**26**]
13	Educate about healthy lifestyle offered in groups with other children with overweight or obesity	[18,**21**,^24^]
14	Offer physical activity in exercise groups with other children with overweight or obesity	[18,**21**,^27^]
15	Ensure enough dosage in number of sessions	[3,**23**,^25^,**26**]
16	Ensure present professional knowledge in healthcare professionals of dietary habits, physical activity, and child-raising	[15,**23**]
17	Ensure sufficient time for preparation of the intervention	[28]
18	Healthcare professionals learn to indicate and discuss successes and obstacles in changing their behaviour.	[14–17,**20**]
19	Healthcare professionals apply suitable theories and methods in behaviour change (relational frame theory, behavioural therapy, systemic and solution-focused treatments like motivational interviewing)	[15,18,27]
20	Create willingness of parents for and reaching of optimal skills and knowledge of parents	[**16,20**,^24^]
21	Learn parents how to support and reinforce children’s healthy weight-related behaviours	[14,**20**]
22	Alternate different ways of delivering, e.g., theory alternated by practical lessons	[18,25]
23	Involve parents and family	[15–18,22,**23,**^27^,^29^]
24	Facilitate positive parent×child interactions	[**30**,**31**]
25	Help parents to understand their infant’s perspective or inner world	[**30**,**31**]
26	Use video feedback	[**30**]
27	Allow one professional to manage the total of support and care (central caregiver), in contact with the child, with the possibility of involving other professionals on an indicative basis	[**23**]
28	Professionals, children and parents set goals jointly	[**23**]
29	Ensure participation of a dietician or nutritional specialist and physician in a therapeutic team	[**26**]
30	Addressing socio-emotional problems	[**16**]
31	Ensure health care professionals are approachable	[**16**]
32	Ensure consistent communication from different health care professionals	[**16**]
33	Appoint a coordinator who has contact with all other professionals involved (not necessarily treating)	[**10**]

*Bold: Result of repeated literature search, 2023.

### Participating experts

Forty-two experts of the 103 invited (42%) participated in the online expert consultation. Seventeen of the 61 experts that had not participated provided reasons for non-participation, which were mainly practical reasons and not related to this study (for example vacation). Although invited experts were asked to forward the invitation when indicated, no additional experts participated. Finally, we included 24 experts in research or evaluation of CLIs, 7 experts in development or coordination of CLIs and 11 experts in the implementation of a CLI participated.

Professors, leading experts in the field of obesity or children with obesity (such as the consultants of the national institute of public health and the environment), authors participating in the latest version of the Dutch guidelines or other relevant articles, researchers, consultants of municipality or public health service and professionals carrying out a CLI, participated.

Researchers were experienced in research or evaluation of CLIs as a researcher associated with an university or research institute. The experts involved in development or coordination worked for example as a policy advisor at a municipality/ public health service. The experts in implementation, for example as a dietitian of physiotherapist, had experience in carrying out a CLI in one or more communities.

### Additions to the literature

Of the 42 participating experts, 16 experts indicated the list of potentially effective components from literature as ‘complete’. Suggestions of the other 26 experts resulted in:

a) eighteen new components, b) five literature suggestions, resulting in seven newly identified components, c) suggestion to change description of four components, and d) five new components by splitting literature-retrieved components.

Three experts were consulted for more details, because the suggested component had similarities with a component already identified from the literature. Consultation resulted in an improved description of components that could be added to the literature overview.

Finally, the 23 potentially effective components we extracted of the literature, the 30 potentially effective components suggested by the experts and the adaptations we made resulted in 53 potentially effective components. See ‘Table 2 Potentially effective components based on literature and expert suggestions’ for the overview of the potentially effective components that we categorised into three themes: content (19 components), organisation (16 components) and implementation (18 components). Of these components, 9 components related to the organisation and 13 components related to the implementation were identified by the experts in addition to the components identified through our literature search.

**Table 2:** Potentially effective components based on literature and expert suggestions.

***THEME*: *ORGANISATION***	***THEME*: *CONTENT***
**From literature:** • Ensure enough dosage in length of sessions • Ensure enough dosage in number of sessions • Offer physical activity in exercise groups with other children with obesity • Offer education about healthy lifestyle in groups with other children with obesity • Involve parents • Ensure present specialized professional training and knowledge of dietary habits, physical activity, and child-raising • Ensure sufficient time for implementors to preparate the intervention • *Allow one professional to manage the total of support and care of the patient (central caregiver)*, *in contact with the child*, *with the possibility of involving other professionals on an indicative basis* • *Let a dietician or nutritional specialist and a physician participate in the team* • *Appoint a coordinator who has contact with all other professionals involved (not necessarily treating)*	**From literature:** • Realise awareness • in children and their parents • Enhance motivation • Discuss the exemplary role of the parent • Support parents in raising children • Adapt content to the culture of the child • Adapt content to the income of parents • Create a tailored treatment plan (that fits • into daily life of the whole family) • Discuss the influence of the physical environment of the child • Discuss the influence of the social environment of the child • Encourage spend less time inactive/watching television • Discuss and optimize sleep patterns • and duration • *Help parents to understand their infant’s perspective or inner world* • *Set goals jointly with the child and parents* • *Address socio-emotional problems*
**From experts:** • CLI is part of (existing) chain care/network • Offer after-care • Offer sessions at home of the child • Offer support of a psychologist • Team of implementors is multidisciplinary • Ensure sufficient cooperation in the team • Prevent unambiguous advice of implementors • Use appropriate information material • Use appropriate location	**From experts**: • Relate behaviour and symptoms • Set small, achievable goals • Accomplish appropriate forms of physical activity that suits the child’s capability’s • Stimulate intrinsic motivation • Discuss how to call upon the network • of the child • Learn how to read and understand • food labels • Visualize (un)healthy food •
***THEME*: *IMPLEMENTATION***	
**From literature**: • Indicate and discuss successes and obstacles in behaviour change • Apply suitable theories and methods in behaviour change • Stimulate the willingness for reaching optimal skills and knowledge • Learn parents how to support and reinforce children’s healthy weight-related behaviour • Alternate different ways of delivering, e.g., theory alternated by practical lessons *Facilitate positive parent×child interactions* *Use video feedback* *Ensure approachable implementors* *Ensure consistent communication from different health care professionals* **From experts**: • Increase the difficulty of tasks • Discuss how barriers can be reduced • Stimulate children to reflect on behaviour goals (self-management) • Stimulate the willingness for • parenting support • Increase the self-confidence of the parents • Take the phase of parents’ behavioural change into account • Take the phase of child’s behavioural change into account • Give positive feedback to the child • Give positive feedback to the parents • Create a positive atmosphere • Stimulate the children of having of fun • Let children support and learn from • each other • Reward children • Use digital media	

*Italics: Result of repeated literature search, 2023.

### Construction of the final checklist

For the development of the final checklist we incorporated the 53 potentially effective components. If applicable, additional answer options were created, like amounts of sessions. In addition, we added space to report for other features like the name of the CLI protocol, user, and date. For most of the potentially effective components, we included answer options present/absent.

To test usability of the final checklist, two researchers used selected CLI protocols. Except for five components, researchers scored similar on the checklist for each CLI protocol. Three components were hard to score due to unclear wording, so descriptions were altered. Two components were split into two separate items because these items contained two constructs, resulting in a total of 55 potentially effective components in the final version of the checklist. The order of components remained unchanged, and all the possible answer options appeared to be present. After evaluating four CLI protocols, saturation was reached. In other words: after assessing four CLI protocols, item description was optimized, two researchers scored exactly the same and the checklist reached its new version. Noteworthy, when testing our pre-final checklist, we found that only a small amount (30%) of the potentially effective components included in our checklist were described in the protocols. These protocols lack components related to connection of the CLI to the neighbourhood, composing and maintaining the network of professionals, and how to involve parents.

### Results repeated literature search

The repeated literature search and synthesis resulted in ten additional potentially effective components. These components are added to: ‘Table 1 Potentially effective components described in literature’. The bold literature indicates the components found by means of the repeated literature search. In addition, we added these components to the overview in ‘Table 2 Potentially effective components based on literature and expert suggestions’. Three components are added to the theme ‘content’, also three components are added to the theme ‘organisation’ and four components are added to the theme ‘implementation’.

Lastly, we incorporated the components found by means of the repeated literature search in our final checklist. After the repeated literature search, the checklist has been extended from 55 to 65 components regarding the content, organisation, and implementation of a CLI.

## Discussion

The aim of this study was to develop a checklist to identify components that may predict successes in CLIs for children with overweight or obesity: potentially effective components. We were able to develop and pilot a practical checklist, based on a literature search, and complemented by consultation of a broad group of experts. The final checklist consists of 65 potentially effective components regarding the content, organisation and implementation of a CLI. As far as we know, this is the first checklist developed on potentially effective components in CLI for children.

An important strength of this study, is that we combined a literature search with opinions of experts in research or evaluation of CLIs, development or coordination of CLIs, or implementation of a CLI. Combining these two allowed us to specify components that are already described in the literature and to add more potentially effective components. While literature, for example, already indicated that a combined treatment for children in which three or more components (nutrition, physical activity, and behaviour) are offered is effective [3,5,6], expert opinions allowed us to further specify this component. For example, experts mentioned: ’a multidisciplinary team of professionals’ and ’support of a psychologist’. Furthermore, regarding the treating team, experts additionally mentioned: ‘sufficient cooperation in the team’ and ‘avoiding unambiguous advice of implementors’. Especially on organizational and implementation level, experts added potentially effective components to our checklist that we would not have found by a literature search only. This shows that the experts made a valuable contribution, especially to the aspects that are important in practice when implementing a CLI.

Since we used literature available in PubMed for the development of our checklist, we may have missed potentially effective components from that could have been retrieved by searching other databases. However, we think that in combination with expert consultations this literature search sufficiently enabled a checklist of potentially effective components. In addition, we are convinced that the knowledge base after our updated search reached a sufficient level of saturation.

For adults with overweight or obesity, three Dutch CLIs have recently been studied for effective components [10]. Also, effective components specific for adults with an extremely increased weight-related health risk [13] and for adults with overweight or obesity with a low social economic status [12] have recently been studied. Many of the effective components in CLIs for adults with overweight or obesity that emerged in these studies are also included in our checklist for CLIs for children, such as: ‘goal setting’ and ‘encouraging self-monitoring’. These components were found for both children and adults. This makes it more likely that these components (regardless of age group) are potentially effective. Therefore, it is appropriate that we added these components to the checklist.

Since there are no comparative studies on the effects of separate components [23], we have no indications which specific components make a greater contribution to the effectiveness of an intervention compared to others. Therefore, the components we retrieved from literature were considered equally important. Also, every expert had an equal share in providing components. As a result, regarding the instrument construction, we have not given weights to separate items or to a combination of components in the checklist. However, in practice, various components are combined in one CLI because a CLI should be adapted to the community and its characteristics [7]. It may well be that certain components are more effective than others, or that a precise combination of components may result in an optimal CLI. This definitely requires further research. To determine the relative contribution of separate items and of a possible combination, and whether giving weight to one or more item(s) is necessary, our checklist must be applied in developing or evaluating different CLIs. In addition, interventions which have been developed in experimental settings are not necessarily suitable for implementation according to protocol in real-life settings, because these settings differ substantially [32,33]. Thus, adaptations are inevitable in the translational process from research to practice [7] and must therefore also be considered when using our checklist to study potentially effective components based on the protocol of a CLI. Until then, as a remark, the components included in the checklist should be considered ‘potentially effective’ and not ‘actually effective’. In addition, it should be kept in mind that we only studied CLI protocols and therefore, absence of a potentially effective component in a protocol does not necessarily mean that the component is not addressed during implementation of the CLI.

We aimed to study potentially effective components for children aged 4–18 years without making any adjustments or stratification on age categories. If within this age category differences exist between for instance children and adolescents, needs to be investigated in further research.

Lastly, the researchers who have developed the checklist were the same as those who have been involved in assessing the usability of the developed checklist (except components extracted by the repeated literature search). Since these researchers already knew the components and background information well, the usability may have been evaluated more positively than it may be evaluated in practice. If the checklist is used in practice, we will monitor this process and we will adjust the checklist if necessary.

Considering all strengths and weaknesses, we believe we have made a good first step towards the identification of potentially effective components of CLIs for children with overweight or obesity. And, although the effect of a CLI is context-dependent [7], we believe that our checklist, due to the combination of literature and expert opinions, is applicable for CLIs in a wide variety of contexts. We recommend the following:

- Use our checklist in developing or evaluating different CLIs and consider all items when developing, evaluating, and adapting CLIs for children with overweight or obesity.
- Complete the checklist with more than one assessor independently of each other and thereafter discuss the scores on item level.
- Complementary to an assessment of the presence of the items in the written protocol of the CLI, monitor the quality of the implementation (’implementation fidelity’). With other words: also analyse the actual implementation. For example, combine the results of this checklist with interviews in practice.
- Identify the value of separate components in predicting outcomes of CLIs. Thereby, also more insight arises into whether it is necessary to add relative weight per item. In addition, attention should be paid to positive interactions of items.
- If within the age category 4–18 years exist differences between for instance children and adolescents is not evaluated and needs to be investigated further.

## Conclusions

A combination of literature and field research has resulted in a checklist of potentially effective components of CLIs for children with overweight or obesity. With the help of this checklist, people working in practice, policy or research can improve the CLIs. By using the developed checklist, the presence of potentially effective components in a CLI that may contribute to positive health effects in practice can be identified. The checklist must be applied to more CLIs to systematically evaluate the value of separate components or specific combination of components.

## Supporting information

S1 Checklist Checklist potential effective components of combined lifestyle interventions for children with overweight or obesity. (PDF)
