# Supplementary material for: Development of a checklist to assess potentially effective components in combined lifestyle interventions for children with overweight or obesity
Source: PLoS One. 2023 Sep 28;18(9):e0289481. doi: 10.1371/journal.pone.0289481 (PMC10538710; doi:10.1371/journal.pone.0289481)
Supplement: S1 Checklist — (PDF) [file pone.0289481.s001.pdf]

Supporting information 1 Checklist

Checklist Potential effective components of combined lifestyle interventions for children with overweight or obesity

This checklist is intended to assess protocols of combined lifestyle interventions (CLIs) for children with overweight or obesity (aged 4 – 18 years), for the presence or absence of potentially effective components.

The list assesses whether a potentially effective component is described in the protocol (also called: script, manual, instruction). It is best to refrain from interpretation.
Therefore, only choose the option "present" if the component is actually described in the protocol.
For items with open questions and multiple-choice options, the most appropriate answer can be entered.
Make sure that the most recent version of the protocol is used.

Name CLI: ____

Name assessor 1: ____

Name assessor 2: ____

Date: ____

Used document (protocol, manual) and version: ________________________________

	Present	Absent
**Theme 1: The content of the CLI**<br>**The protocol takes into account …**		
the **relationship between** health behavior and symptoms		
**awareness is realized** in children and their parents		
stimulation of the **intrinsic motivation** of the child		
stimulation of the **extrinsic motivation** of the child		
**socio-emotional problems** are addressed		
the exemplary **role of the parent**		
**child-raising**		
professionals helping parents **to understand their infant's perspective or inner world**		
**the cultural background** of the child		
the **income of parents**		
setting **small, achievable goals**		
the treatment plan **fits into daily life** of the whole family		
**limiting sedentary behavior**		
an **appropriate form of physical activity** for the child is searched		
the **influence of physical environment** on the child's health behavior		
the **influence of social environment** on the child's health behavior		
**dealing with social pressure and support**		
supporting of the child and their parents by family, friends and other people **from their immediate environment and network**		
the importance of the **topic sleep**		
**improvement of sleep patterns and duration**		
**reading and understand nutrition labelling**		
**visualization** of (un)healthy food		

**Theme 2: Organizational aspects**<br>**The protocol describes…**	**Present**	**Absent**
that the CLI is part of **chain care**		
**A coordinating caregiver** is appointed who has contact with all other professionals involved (not necessarily treating)		
**One professional manage the total of support and care** of the patient (central caregiver), in contact with the child, with the possibility of involving other professionals on an indicative basis		
the total **duration** of the CLI<br>*If present, total duration:* ______.		
**after-care or follow up** time<br>*If present, duration:* .______.		
the number of **group sessions** with other children:<br>*If present, amount nutrition sessions:* .______.<br>*If present, amount physical activity sessions:* .______.<br>*If present, amount mental health sessions:* .______.<br>*If present, amount other sessions:* .______.		
the number of **individual sessions** with implementor(s):<br>*If present, amount nutrition sessions:* .______.<br>*If present, amount physical activity sessions:* .______.<br>*If present, amount mental health sessions:* .______.<br>*If present, amount other sessions:* .______.		
**home visit(s)** of the involved implementor(s)<br>*If present, which and amount:* .______.<br>.______.		
**more than one discipline** is involved in the implementation<br>*If so, which disciplines*: .______.<br>.______.		
**support of a psychologist** is offered		
**support of a dietician or nutritional specialist** is offered		
requirements for the qualifications or the **level of education** of the implementors<br>*If present, which:* .______.<br>.______.		
**contact-moments** between the different implementors<br>*If present, frequency:* .______.<br>.______.		
**time** for implementors to prepare the sessions<br>*If present, time:*.______.		
attention to avoid that implementors give **ambiguous advice**		

that **family is invited** to attend one or more meetings<br>*If present, amount:* ________________________________		
**requirements for the number** of CLI meetings that the family must attend<br>*If present, which:* ________________________________		
that used **material is specifically aimed** for children		
that sessions take place in a **location that is attractive** to children		
**Theme 3: Implementation**<br>**The protocol describes to…**	**Present**	**Absent**
pay attention to **increase the difficulty** of tasks for behavior change		
**Set goals jointly** with the child and parents		
indicate and discuss **successes and obstacles** in changing behavior		
discuss how experienced **barriers can be reduced**		
stimulate children to **reflect on behavioral goals (self-management)**		
**apply suitable theories and methods** in behavior change<br>(like behavioral therapy, motivational interviewing)		
stimulate the willingness for **reaching optimal skills and knowledge in parents**		
stimulate the willingness for **parenting support**		
learn parents **how to support and reinforce** children's healthy weight-related behavior		
facilitate **positive parent × child interactions**		
pay attention to **increase the self-confidence** of the parent(s)		
**take the phase** of <u>parents'</u> behavioral change into account		
**take the phase** of <u>child's</u> behavioral change into account		
stimulate desired behavior of the <u>child</u> by **giving positive feedback**		
stimulate desired behavior of the <u>parents</u> by **giving positive feedback**		
pay attention to a **positive atmosphere** in the group sessions		
facilitate that **children have fun** during the treatment		
children **support each other and learn** from each other		
ensure **implementors are approachable**		
ensure **consistent communication** from different health care professionals		

stimulate desired behavior by **giving rewards**		
alternate **different ways of delivering**,<br>e.g., theory alternated by practical lessons and instructions		
use **digital media**		
use **video feedback**		
